# Response of an aggressive periosteal aneurysmal bone cyst (ABC) of the radius to denosumab therapy

**DOI:** 10.1186/1477-7819-12-17

**Published:** 2014-01-20

**Authors:** Chantal Pauli, Bruno Fuchs, Christian Pfirrmann, Julia A Bridge, Silvia Hofer, Beata Bode

**Affiliations:** 1 Institute of Surgical Pathology, University Hospital Zurich, CH-8091, Zurich, Switzerland; 2 Orthopedics, University Hospital Balgrist, Zurich, Switzerland; 3 Radiology, University Hospital Balgrist, Zurich, Switzerland; 4 Department of Pathology/Microbiology, University of Nebraska Medical Center, Omaha, NE, USA; 5 Oncology, University Hospital Zurich, Zurich, Switzerland

**Keywords:** Aneurysmal bone cyst, FISH, USP6, Denosumab

## Abstract

Aneurysmal bone cyst (ABC), once considered a reactive lesion, has been proven to be a neoplasia characterized by rearrangements of the *USP6*-gene. Aggressive local growth and recurrences are common and therapeutic options may be limited due to the vicinity of crucial structures. We describe a case of a locally aggressive, multinucleated giant cell-containing lesion of the forearm of a 21-year old woman, treated with denosumab for recurrent, surgically uncontrollable disease. Under the influence of this RANKL inhibitor, the tumor showed a marked reduction of the content of the osteoclastic giant cells and an extensive metaplastic osteoid production leading to the bony containment, mostly located intracortically in the proximal radius. The diagnosis of a periosteal ABC was confirmed by FISH demonstrating *USP6* gene rearrangement on the initial biopsy. Function conserving surgery could be performed, enabling reconstruction of the affected bone. Inhibition of RANKL with denosumab may offer therapeutic option for patients not only with giant cell tumors but also with ABCs.

## Background

Aneurysmal bone cysts (ABCs) are rare benign skeletal tumors usually occurring in the first two decades of life and typically associated with a growing mass, swelling, pain and bone destruction [1,2]. Approximately 70% of the cases are considered primary lesions and the remaining 30% arising secondary to different primary tumors such as osteoblastoma, giant cell tumor of bone, chondroblastoma, fibrous dysplasia or low grade intramedullary osteosarcoma [3]. It has been shown that primary ABCs, initially considered to be reactive and non-neoplastic [1,4], correspond to a neoplastic disorder associated with a specific set of genetic aberrations [5,6]. Over the past few years many different translocations have been described in ABCs, all resulting in oncogenic activation of the *USP6* gene localized to 17p13 [7-9].

Primary ABCs can affect any bone. Usually they arise in the metaphysis of long bones such as the femur, tibia and humerus; however, the spine may also be affected with compression/infiltration of nerves and spinal cord causing neurological symptoms [10]. The magnetic resonance imaging (MRI) demonstrates an intraosseous, expansile, lytic, eccentric, septated lesion, containing characteristic fluid-fluid levels, except for rare solid ABC variants. Rarely, the ABC is localized on the surface of the long bones [11]. Histopathologically, the lesions are composed of hemorrhagic tissue with cavitary spaces separated by fibrous septa composed of spindle cells, inflammatory cells and a smaller percentage of giant cells [12]. Treatment options are intralesional curettage followed by bone grafting, in combination with cryotherapy, sclerotherapy, radionuclide ablation, arterial embolization and *en bloc* resection [13,14]. Complications associated with curettage are related to an incomplete resectability of the lesion resulting in recurrence in at least 20% [2]. Clinically [15], ABCs can be divided into inactive, active and aggressive lesions with aggressive tumors expanding rapidly, destroying surrounding tissue and having a high rate of local recurrences. New therapeutic options are needed for the management of this locally aggressive disease.

Denosumab, a monoclonal antibody specifically binding RANK-ligand, inhibits bone resorption and, therefore, [16-18] is used in the treatment of osteoporosis, skeletal complications of metastatic disease, and more recently in the treatment of giant cell tumors of bone, with a high rate of clinical success [19]. To date, we are aware of only one publication presenting the application of denosumab in two cases of spinal ABCs [20]. Both patients (an 8-year old boy and an 11-year old boy) recovered significantly from pain and neurological symptoms. MRI follow-up after two to four months of denosumab therapy showed tumor regression in both patients. We report a case of a locally aggressive periosteal ABC with a confirmed rearrangement of *USP6* arising in the radius of a 21-year old woman with an impressive local response to denosumab treatment and a follow-up of four years.

## Case presentation

**Figure 1:**
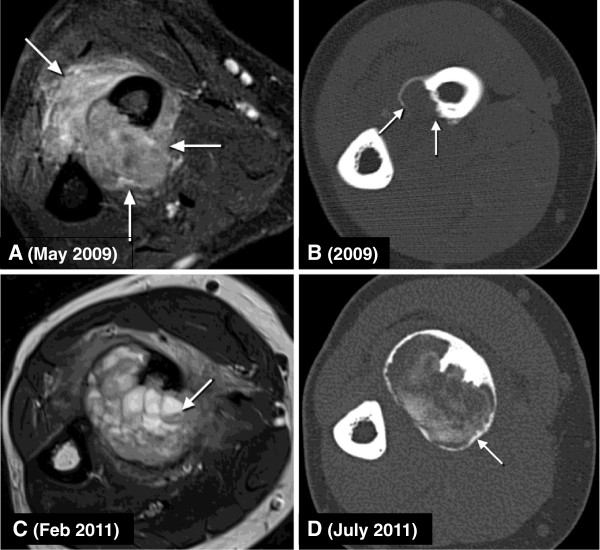
**Imaging of the patients right forearm tumor. (A)** Initial magnetic resonance imaging (MRI) demonstrating extensive involvement of the soft tissue between the radius and ulna as well as the cortex of the radius by an exclusively solid tumor mass (arrows). **(B)** Pre-treatment computer tomography (CT) scan with a small area of a split and disrupted cortex of the radius (arrows). **(C)** MRI directly prior to denosumab therapy with a locally progressive, extensive soft tissue mass following local surgical therapy 18 months previously. Fluid-fluid levels may be seen at this point (arrow). **(D)** CT scan following five months of denosumab therapy demonstrating almost complete containment of the soft tissue mass by a boney rim (arrow).

**Figure 2:**
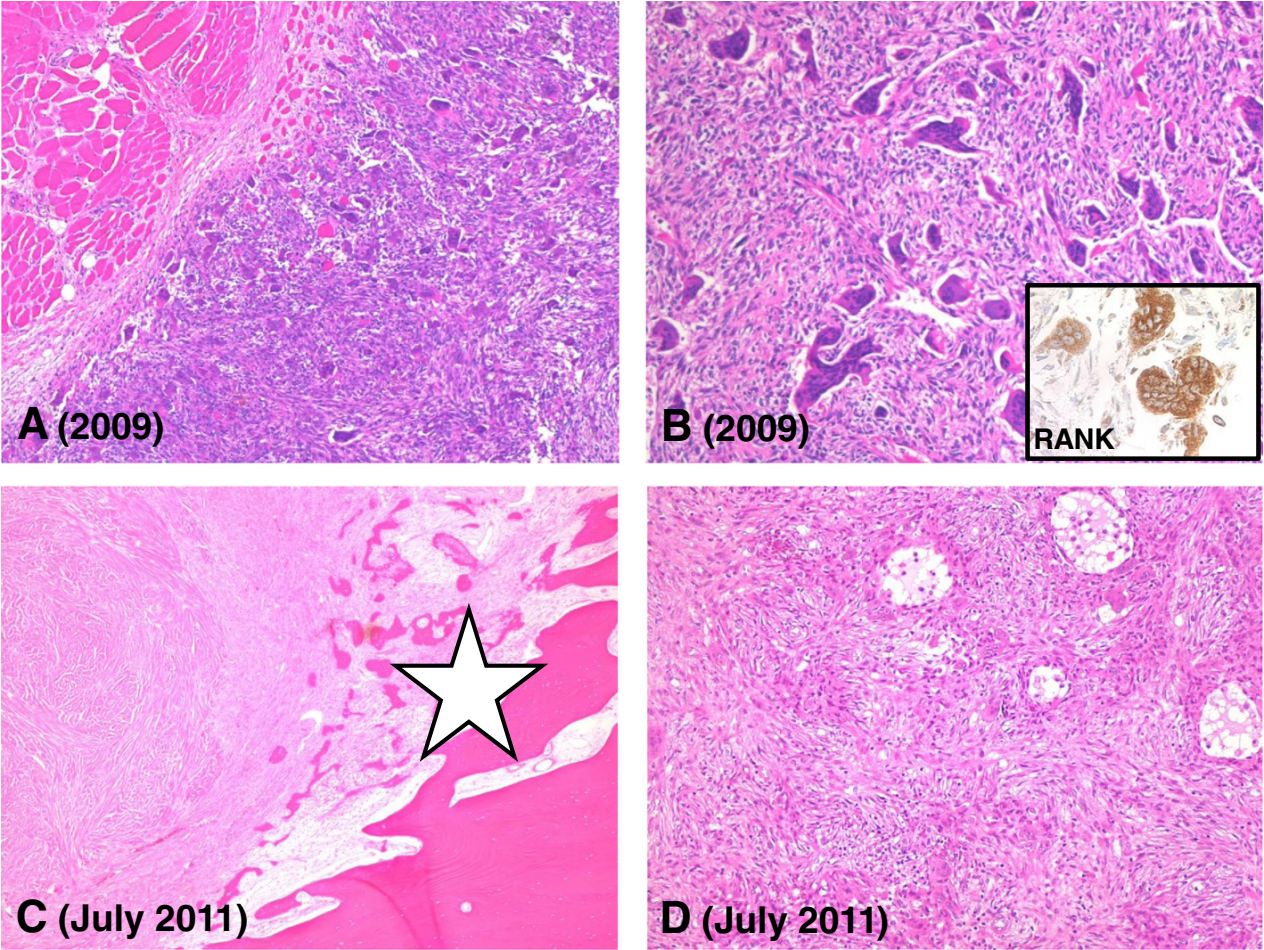
**Histopathology of the pre- and post-treatment tumor tissue specimen. (A)** Pre-treatment biopsy sample showing giant cell containing soft tissue mass with extensive infiltration of the skeletal muscle (H&E stain; original magnification 50×). **(B)** Abundant lesional giant cells with numerous nuclei and mononuclear cells in the background (H&E stain; original magnification 100×). Inset shows immunohistochemical expression of RANK (dilution 1:400; R&D Systems, Abingdon, United Kingdom) by the osteoclastic giant cells. **(C)** Denosumab treatment induced boney containment (asterisk) of the tumor (upper left) (H&E stain; original magnification 25×). **(D)** Post-denosumab-treatment tumor specimen showing pronounced reduction of the number of giant cells (H&E stain; original magnification 100×).

A 21-year old right-handed woman presented with a variable swelling and shooting pain in her right proximal forearm in May of 2009. Clinical examination showed a palpable swelling over the radial head mainly located over the biceps tendon and a supination deficiency. MRI revealed an extensive, deep seated, solid soft tissue tumor with contrast uptake, infiltration of the intra-osseous membranes, biceps tendon, contact with the neurovascular bundle, infiltration of the supinator muscle and deep extensor as well as deep flexor muscles (Figure 1A, B). Computer tomography (CT)-guided core-needle biopsy was performed with a clinical suspicion of Ewing sarcoma. A low-grade, giant cell-containing lesion with focal metaplastic bone formation and infiltration of the skeletal muscle was diagnosed on histopathological examination (Figure 2A). No necrosis, atypia or pathologic mitotic activity was noted. The osteoclastic giant cells were numerous and contained up to over 50 nuclei.

**Figure 3:**
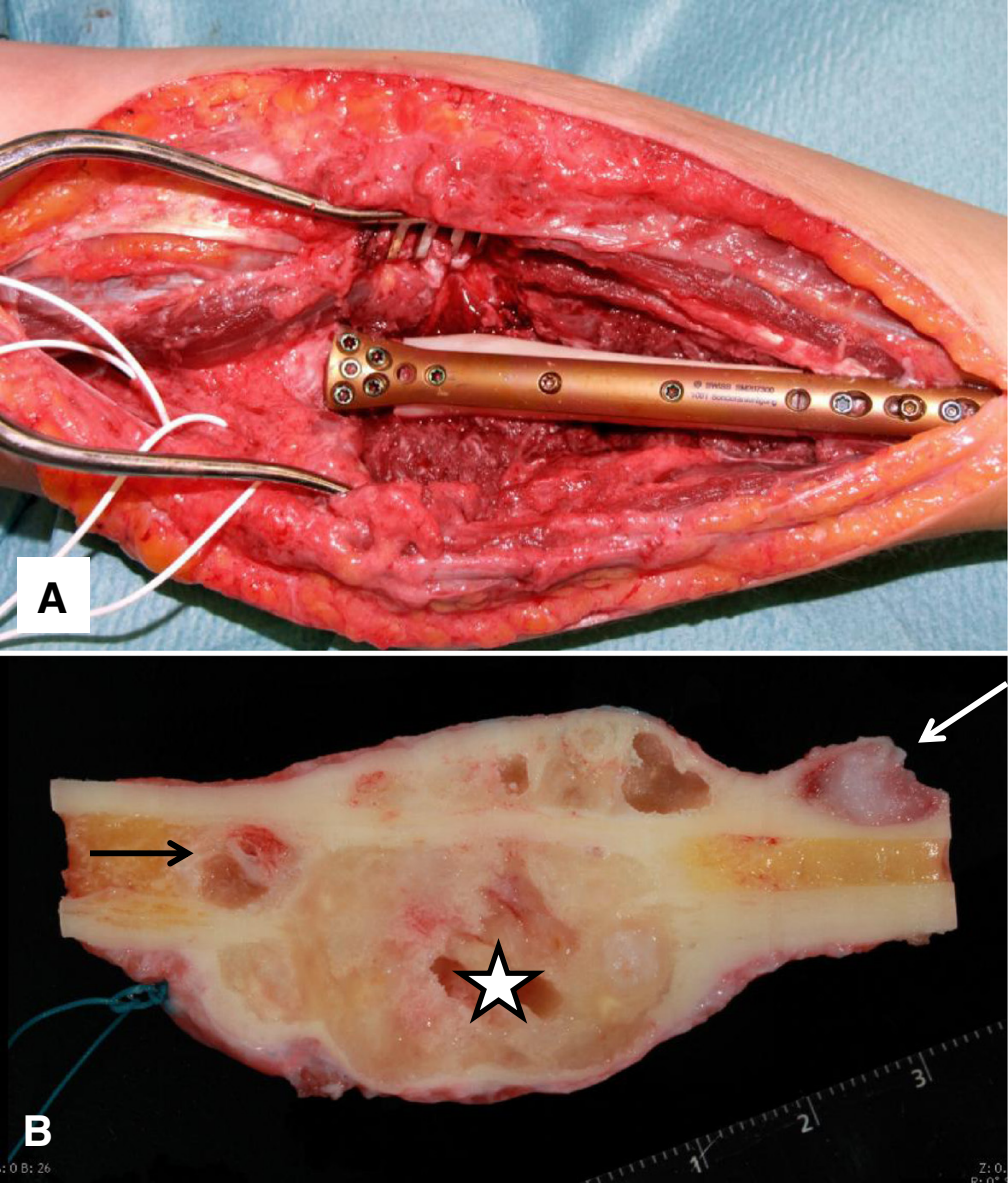
**Resection of the shaft of the proximal radius following the denosumab therapy. (A)** Reconstruction using an intercalary fibula-allograft and a custom-made plate. **(B)** Longitudinal section of the resected specimen demonstrating large intracortical, fully contained by a rim of bone tumor mass (asterisk) with a small intramedullar tumor nodule proximally (black arrow) and an intracortical satellite distally (white arrow).

Surgical therapy two months later consisted of a curettage through an antero-lateral and dorsal incision with dissection of the radial nerve branches to preserve maximal function. Histology of the recovered fragmented tumor revealed similar results to the biopsy (Figure 2B) rendering the diagnosis of an incompletely resected giant cell tumor of soft tissue. Residual tumor has been followed clinically and on imaging with a new local progression noticed at the end of 2010. MRI in February 2011 (Figure 1C) showed a significant increase in the size of a recurrent and progressively symptomatic tumor. Under the assumption of the diagnosis of a giant cell tumor of soft tissue, the therapy with denosumab (120 mg subcutaneously injected every month) for four months was administered with no untoward side effects. Follow-up MRI after one month of denosumab showed obvious tumor regression in size. Five months after the first denosumab injection, surgery was performed (July 2011). As a result of good treatment response, the tumor had become significantly better circumscribed and clearly demarcated by a bony rim (Figure 1D), leading to the feasibility of a complete resection of the tumor. Reconstruction was performed using an intercalary fibula-allograft with 3D planning to adjust for radial head rotation and a custom-made plate (Figure 3A). The gross examination of the resection specimen (Figure 3B) revealed an extensive, almost circumferential, intracortical bone tumor mass extending over 4.4 cm of the diaphysis of the radius with a small intramedullary nodule proximally and an intracortical satellite lesion distally. The histology of the post-treatment tumor specimen (Figure 2C, D) differed in comparison to the material of the core biopsy and curettage (Figure 2A, B) showing markedly reduced numbers of osteoclasts. The individual giant cells contained only few nuclei. Few small, pseudocystic spaces were observed in the background of collagen rich connective tissue, containing bland mononuclear cells. There was pronounced metaplastic new bone production with extensive areas of so-called ‘blue bone’ and lamellar cortical bone at the periphery of the lesion (asterisk Figure 2C). Due to the gross findings with almost exclusive intracortical tumor location, the lesion was re-classified as a predominantly intracortical, aneurysmal bone cyst of the proximal radius. Bone and soft tissue resection margins were free of tumor except for an unclear area at the distal corticalis.

**Figure 4:**
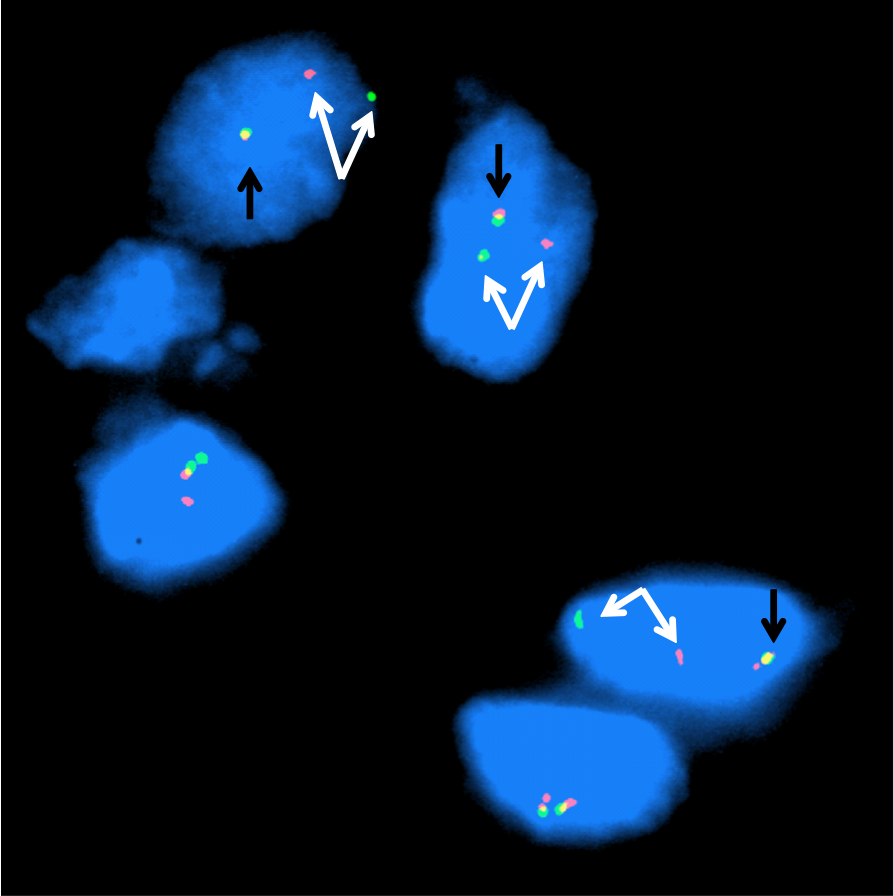
**Fluorescence *in situ *hybridization conducted on the pretreatment specimen with a custom-designed break apart probe set; the probe cocktail proximal to the *USP6 *locus is labeled in green and distal in red.** The mononuclear nuclei exhibit one fused red/green signal corresponding to a normal 17p *USP6* locus (black arrow) and a pair of split green and red signals (white arrows) indicating a rearrangement of the *USP6* locus consistent with the diagnosis of aneurysmal bone cyst.

At first presentation in 2009, most of the exclusively solid tumor mass was localized in the soft tissues of the forearm on imaging (Figure 1A), leading to the diagnosis of giant tumor of soft tissue. Retrospectively, careful analysis of the initial CT scans (Figure 1B) showed an area of split and disrupted cortical bone of the radius, which might have initially suggested the diagnosis of an intracortical (solid variant) ABC with a massive extension to the periosteal tissue. Finally, the diagnosis of a primary ABC was definitively confirmed by demonstration of a rearrangement of the *USP6* gene locus by fluorescence *in situ* hybridization (FISH) as described previously [21], utilizing a custom-designed break apart probe set on the original biopsy sample (Figure 4). The mononuclear nuclei exhibit one fused red/green signal corresponding to a normal 17p *USP6* locus (black arrow) and a pair of split green and red signals (white arrows) indicating a rearrangement of the *USP6* locus consistent with the diagnosis of aneurysmal bone cyst.

Further follow up was unremarkable for another 19 months until a control MRI in February 2013 revealed a small (< 1 cm) lesion in the area of the distal osteotomy and the core biopsy confirmed the local recurrence of the ABC, which is currently being treated with denosumab.

## Conclusion

In this report, we describe a patient treated with denosumab for a locally aggressive, presumed giant cell tumor of soft tissue of the forearm. Evaluation of the resection specimen led to the final diagnosis of an intracortical aneurysmal bone cyst with massive infiltration of the soft tissue, a diagnosis which was also confirmed by FISH analysis.

Histopathologic differential diagnosis of osteoclastic giant cell containing lesions of skeletal tissues is extensive (giant cell tumor of bone, giant cell tumor of soft tissue, tenosynovial giant cell tumor, aneurysmal bone cyst, reparative giant cell granuloma, brown tumor of hyperparathyroidism, non-ossifying fibroma) and the final diagnosis has to be made in close conjunction with clinical and radiological information. Giant cells occurring in both giant cell tumors (GCTs) and in ABCs are positive for markers of true osteoclasts, expressing RANK (receptor activator of NF-κB) and promoting bone destruction [22]. The formation of osteoclast type of giant cells in the GCTs is considered to be a consequence of the local production of RANKL (RANK-ligand) by the mononuclear, true neoplastic tumor cells [23]. A current therapeutic option for patients with giant cell tumors of bone is curettage with optimal preservation of function. However, patients undergoing curettage often have recurrent disease or significant morbidity. Denosumab is a human anti-RANKL monoclonal antibody that inhibits RANKL function and therefore its osteoclast activity, and causes the repression of osteoclast recruitment, maturation and bone resorption [16,18]. Denosumab is a Food and Drug Administration (FDA)-approved drug for osteoporosis and skeletal-related events in patients with bone metastases. A study to explore the action of denosumab in the treatment of giant cell tumor of bone was performed [19], demonstrating that the denosumab treated patients had a decrease in giant cells of 90 percent or greater, an indicator for the reduction of the aggressiveness of the tumors. The application of denosumab for bone tumors other than GCT has rarely been investigated. There exists only one report of denosumab use for ABC in two patients with spinal tumors [20]. However, the molecular confirmation of the primary ABCs as well as the histologic changes observed during and following therapy has not been previously reported.

Denosumab therapy was well tolerated by the current patient. The impressive positive treatment effects on the tumor tissue, as illustrated by marked reduction in the number of giant cells, were coupled with the bony demarcation of the tumor and facilitated subsequent function preserving surgery. The application of this form of the RANKL inhibition led to the transition of a locally aggressive and destructive tumor into a resectable lesion, thus preserving full function in our patient.

Our observations support the hypothesis that not only patients with GCTs, but also those suffering from ABCs may benefit substantially from RANKL-inhibiting denosumab treatment. These findings may offer new therapeutic options for often young patients with these locally aggressive tumors, especially in uncontrollable, locally destructive or recurrent disease.

## Consent

Written informed consent was obtained from the patient for the publication of this report and any accompanying images.

## Abbreviations

ABC: Aneurysmal bone cyst; CT: Computer tomography; FISH: Fluorescence *in situ* hybridization; GCT: Giant cell tumor; H&E: Hematoxylin and eosin; MRI: Magnetic resonance imaging; RANK: Receptor activator of NF-κ; RANKL: Receptor activator of NF-κ ligand; USP6: Ubiquitin carboxyl-terminal hydrolase 6.

## Competing interests

The authors indicate no potential conflict of interests.

## Authors’ contributions

CP and BB performed histopathologicals studies and drafted the manuscript. BF and SH coordinated the management and the treatment of the patient, concerning the surgeries and denosumab treatment respectively. CP coordinated the imaging studies. JB carried out the molecular genetic studies. All authors read and approved the final manuscript.
